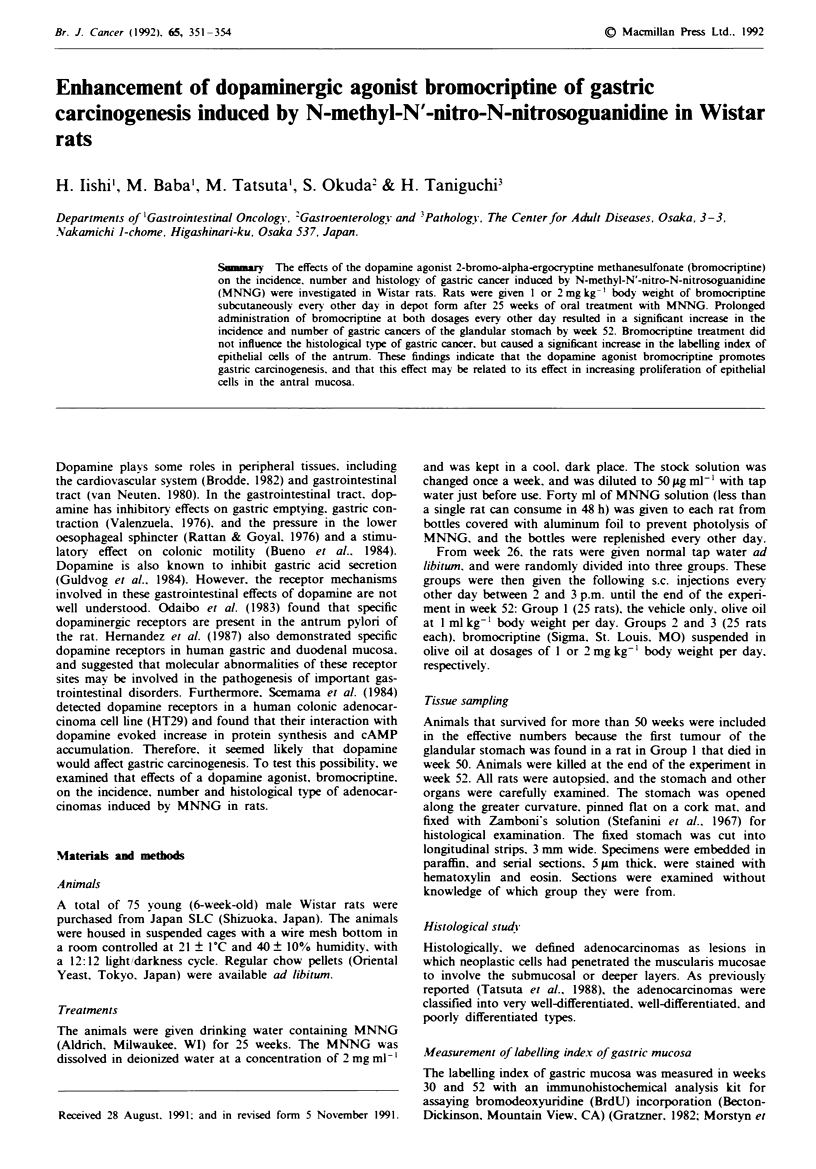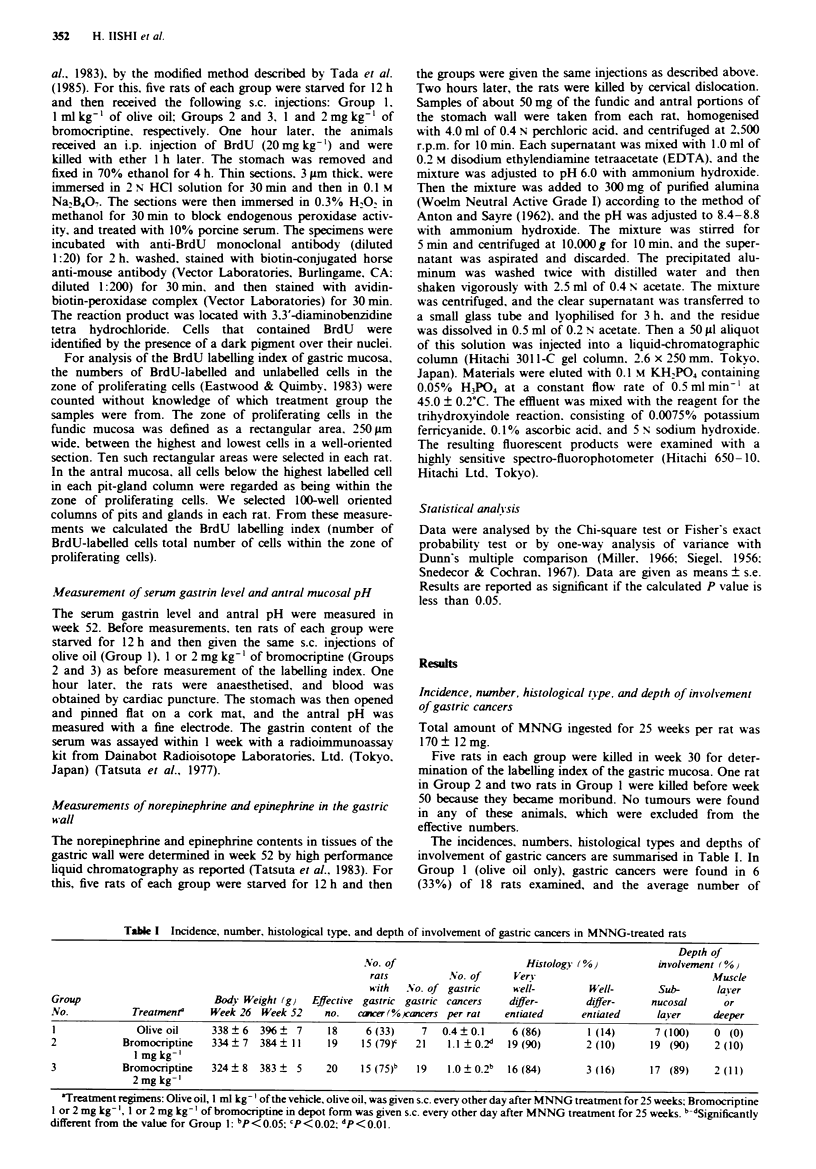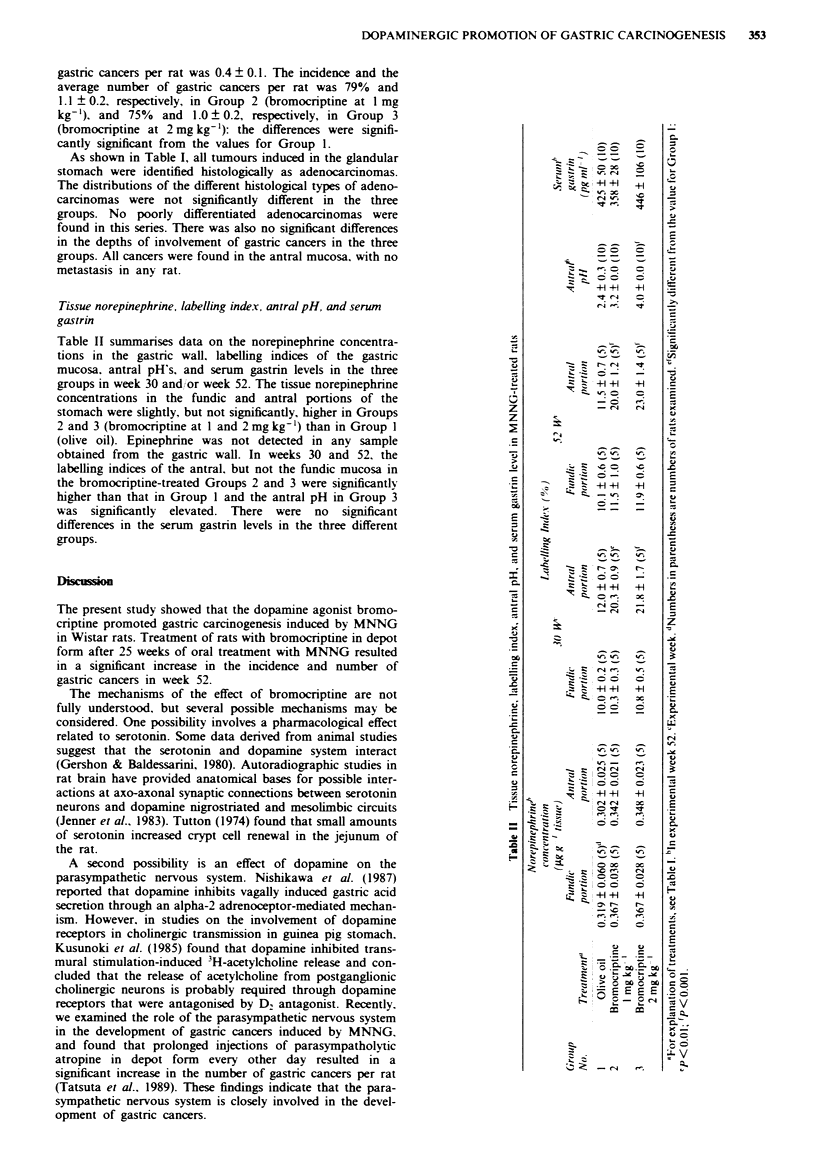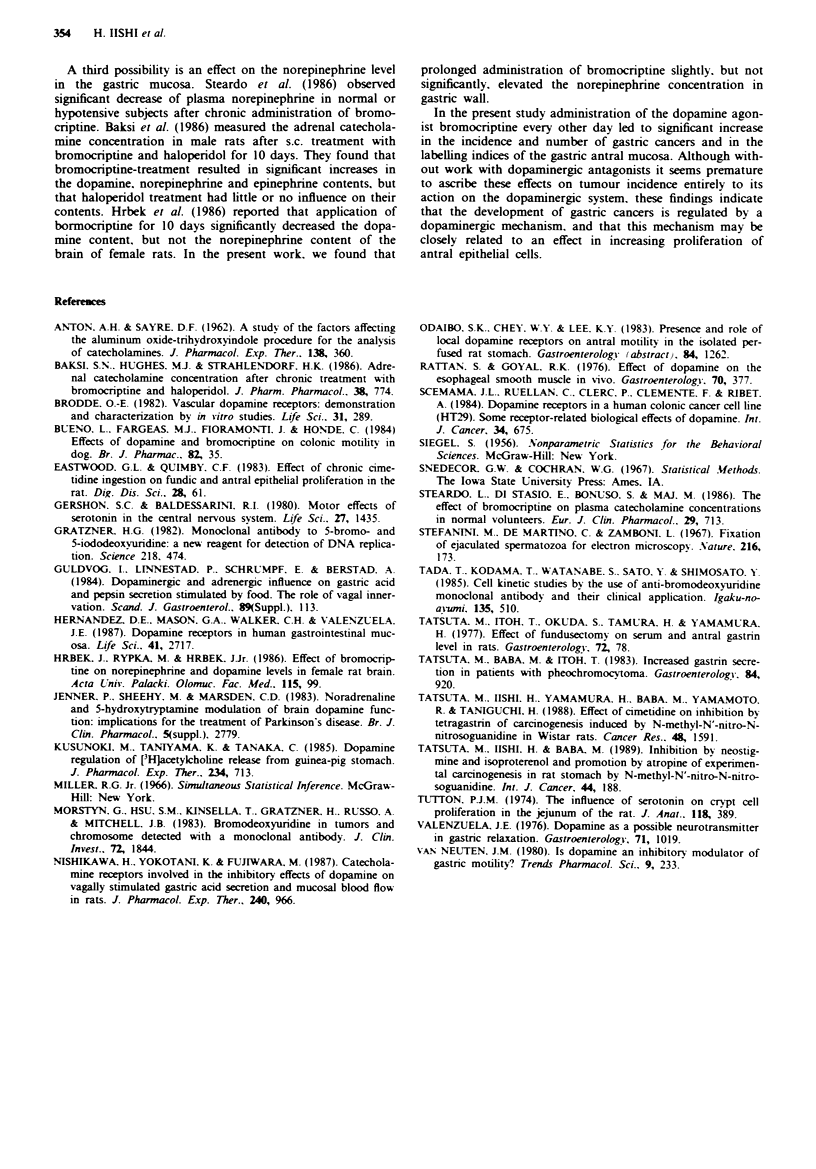# Enhancement of dopaminergic agonist bromocriptine of gastric carcinogenesis induced by N-methyl-N'-nitro-N-nitrosoguanidine in Wistar rats.

**DOI:** 10.1038/bjc.1992.71

**Published:** 1992-03

**Authors:** H. Iishi, M. Baba, M. Tatsuta, S. Okuda, H. Taniguchi

**Affiliations:** Department of Gastrointestinal Oncology, Center for Adult Diseases, Osaka, Japan.

## Abstract

The effects of the dopamine agonist 2-bromo-alpha-ergocryptine methanesulfonate (bromocriptine) on the incidence, number and histology of gastric cancer induced by N-methyl-N'-nitro-N-nitrosoguanidine (MNNG) were investigated in Wistar rats. Rats were given 1 or 2 mg kg-1 body weight of bromocriptine subcutaneously every other day in depot form after 25 weeks of oral treatment with MNNG. Prolonged administration of bromocriptine at both dosages every other day resulted in a significant increase in the incidence and number of gastric cancers of the glandular stomach by week 52. Bromocriptine treatment did not influence the histological type of gastric cancer, but caused a significant increase in the labelling index of epithelial cells of the antrum. These findings indicate that the dopamine agonist bromocriptine promotes gastric carcinogenesis, and that this effect may be related to its effect in increasing proliferation of epithelial cells in the antral mucosa.


Br. J. Cancer (1992), 65, 351-354 © Macmillan Press Ltd., 1992

### Enhancement of dopaminergic agonist bromocriptine of gastric carcinogenesis induced by N-methyl-N′-nitro-N-nitrosoguanidine in Wistar rats

H. Iishi[1], M. Baba[1], M. Tatsuta[1], S. Okuda[2] & H. Taniguchi[3]

*Departments of [1]Gastrointestinal Oncology, [2]Gastroenterology and [3]Pathology, The Center for Adult Diseases, Osaka, 3-3, Nakamichi 1-chome, Higashinari-ku, Osaka 537, Japan.*

**Summary** The effects of the dopamine agonist 2-bromo-alpha-ergocryptine methanesulfonate (bromocriptine) on the incidence, number and histology of gastric cancer induced by N-methyl-N′-nitro-N-nitrosoguanidine (MNNG) were investigated in Wistar rats. Rats were given 1 or 2 mg kg$^{-1}$ body weight of bromocriptine subcutaneously every other day in depot form after 25 weeks of oral treatment with MNNG. Prolonged administration of bromocriptine at both dosages every other day resulted in a significant increase in the incidence and number of gastric cancers of the glandular stomach by week 52. Bromocriptine treatment did not influence the histological type of gastric cancer, but caused a significant increase in the labelling index of epithelial cells of the antrum. These findings indicate that the dopamine agonist bromocriptine promotes gastric carcinogenesis, and that this effect may be related to its effect in increasing proliferation of epithelial cells in the antral mucosa.

Dopamine plays some roles in peripheral tissues, including the cardiovascular system (Brodde, 1982) and gastrointestinal tract (van Neuten, 1980). In the gastrointestinal tract, dopamine has inhibitory effects on gastric emptying, gastric contraction (Valenzuela, 1976), and the pressure in the lower oesophageal sphincter (Rattan & Goyal, 1976) and a stimulatory effect on colonic motility (Bueno *et al.*, 1984). Dopamine is also known to inhibit gastric acid secretion (Guldvog *et al.*, 1984). However, the receptor mechanisms involved in these gastrointestinal effects of dopamine are not well understood. Odaibo *et al.* (1983) found that specific dopaminergic receptors are present in the antrum pylori of the rat. Hernandez *et al.* (1987) also demonstrated specific dopamine receptors in human gastric and duodenal mucosa, and suggested that molecular abnormalities of these receptor sites may be involved in the pathogenesis of important gastrointestinal disorders. Furthermore, Scemama *et al.* (1984) detected dopamine receptors in a human colonic adenocarcinoma cell line (HT29) and found that their interaction with dopamine evoked increase in protein synthesis and cAMP accumulation. Therefore, it seemed likely that dopamine would affect gastric carcinogenesis. To test this possibility, we examined that effects of a dopamine agonist, bromocriptine, on the incidence, number and histological type of adenocarcinomas induced by MNNG in rats.

### Materials and methods

### *Animals*

A total of 75 young (6-week-old) male Wistar rats were purchased from Japan SLC (Shizuoka, Japan). The animals were housed in suspended cages with a wire mesh bottom in a room controlled at 21 ± 1°C and 40 ± 10% humidity, with a 12:12 light/darkness cycle. Regular chow pellets (Oriental Yeast, Tokyo, Japan) were available *ad libitum*.

### *Treatments*

The animals were given drinking water containing MNNG (Aldrich, Milwaukee, WI) for 25 weeks. The MNNG was dissolved in deionized water at a concentration of 2 mg ml$^{-1}$ and was kept in a cool, dark place. The stock solution was changed once a week, and was diluted to 50 μg ml$^{-1}$ with tap water just before use. Forty ml of MNNG solution (less than a single rat can consume in 48 h) was given to each rat from bottles covered with aluminum foil to prevent photolysis of MNNG, and the bottles were replenished every other day.

From week 26, the rats were given normal tap water *ad libitum*, and were randomly divided into three groups. These groups were then given the following s.c. injections every other day between 2 and 3 p.m. until the end of the experiment in week 52: Group 1 (25 rats), the vehicle only, olive oil at 1 ml kg$^{-1}$ body weight per day. Groups 2 and 3 (25 rats each), bromocriptine (Sigma, St. Louis, MO) suspended in olive oil at dosages of 1 or 2 mg kg$^{-1}$ body weight per day, respectively.

### *Tissue sampling*

Animals that survived for more than 50 weeks were included in the effective numbers because the first tumour of the glandular stomach was found in a rat in Group 1 that died in week 50. Animals were killed at the end of the experiment in week 52. All rats were autopsied, and the stomach and other organs were carefully examined. The stomach was opened along the greater curvature, pinned flat on a cork mat, and fixed with Zamboni's solution (Stefanini *et al.*, 1967) for histological examination. The fixed stomach was cut into longitudinal strips, 3 mm wide. Specimens were embedded in paraffin, and serial sections, 5 μm thick, were stained with hematoxylin and eosin. Sections were examined without knowledge of which group they were from.

### *Histological study*

Histologically, we defined adenocarcinomas as lesions in which neoplastic cells had penetrated the muscularis mucosae to involve the submucosal or deeper layers. As previously reported (Tatsuta *et al.*, 1988), the adenocarcinomas were classified into very well-differentiated, well-differentiated, and poorly differentiated types.

### *Measurement of labelling index of gastric mucosa*

The labelling index of gastric mucosa was measured in weeks 30 and 52 with an immunohistochemical analysis kit for assaying bromodeoxyuridine (BrdU) incorporation (Becton-Dickinson, Mountain View, CA) (Gratzner, 1982; Morstyn *et*

Received 28 August, 1991; and in revised form 5 November 1991.

*al.*, 1983), by the modified method described by Tada *et al.* (1985). For this, five rats of each group were starved for 12 h and then received the following s.c. injections: Group 1, 1 ml $kg^{-1}$ of olive oil; Groups 2 and 3, 1 and 2 mg $kg^{-1}$ of bromocriptine, respectively. One hour later, the animals received an i.p. injection of BrdU (20 mg $kg^{-1}$) and were killed with ether 1 h later. The stomach was removed and fixed in 70% ethanol for 4 h. Thin sections, 3 μm thick, were immersed in 2 N HCl solution for 30 min and then in 0.1 M $Na_2B_4O_7$. The sections were then immersed in 0.3% $H_2O_2$ in methanol for 30 min to block endogenous peroxidase activity, and treated with 10% porcine serum. The specimens were incubated with anti-BrdU monoclonal antibody (diluted 1:20) for 2 h, washed, stained with biotin-conjugated horse anti-mouse antibody (Vector Laboratories, Burlingame, CA; diluted 1:200) for 30 min, and then stained with avidin-biotin-peroxidase complex (Vector Laboratories) for 30 min. The reaction product was located with 3,3′-diaminobenzidine tetra hydrochloride. Cells that contained BrdU were identified by the presence of a dark pigment over their nuclei.

For analysis of the BrdU labelling index of gastric mucosa, the numbers of BrdU-labelled and unlabelled cells in the zone of proliferating cells (Eastwood & Quimby, 1983) were counted without knowledge of which treatment group the samples were from. The zone of proliferating cells in the fundic mucosa was defined as a rectangular area, 250 μm wide, between the highest and lowest cells in a well-oriented section. Ten such rectangular areas were selected in each rat. In the antral mucosa, all cells below the highest labelled cell in each pit-gland column were regarded as being within the zone of proliferating cells. We selected 100-well oriented columns of pits and glands in each rat. From these measurements we calculated the BrdU labelling index (number of BrdU-labelled cells total number of cells within the zone of proliferating cells).

### *Measurement of serum gastrin level and antral mucosal pH*

The serum gastrin level and antral pH were measured in week 52. Before measurements, ten rats of each group were starved for 12 h and then given the same s.c. injections of olive oil (Group 1), 1 or 2 mg $kg^{-1}$ of bromocriptine (Groups 2 and 3) as before measurement of the labelling index. One hour later, the rats were anaesthetised, and blood was obtained by cardiac puncture. The stomach was then opened and pinned flat on a cork mat, and the antral pH was measured with a fine electrode. The gastrin content of the serum was assayed within 1 week with a radioimmunoassay kit from Dainabot Radioisotope Laboratories, Ltd. (Tokyo, Japan) (Tatsuta *et al.*, 1977).

### *Measurements of norepinephrine and epinephrine in the gastric wall*

The norepinephrine and epinephrine contents in tissues of the gastric wall were determined in week 52 by high performance liquid chromatography as reported (Tatsuta *et al.*, 1983). For this, five rats of each group were starved for 12 h and then the groups were given the same injections as described above. Two hours later, the rats were killed by cervical dislocation. Samples of about 50 mg of the fundic and antral portions of the stomach wall were taken from each rat, homogenised with 4.0 ml of 0.4 N perchloric acid, and centrifuged at 2,500 r.p.m. for 10 min. Each supernatant was mixed with 1.0 ml of 0.2 M disodium ethylendiamine tetraacetate (EDTA), and the mixture was adjusted to pH 6.0 with ammonium hydroxide. Then the mixture was added to 300 mg of purified alumina (Woelm Neutral Active Grade I) according to the method of Anton and Sayre (1962), and the pH was adjusted to 8.4–8.8 with ammonium hydroxide. The mixture was stirred for 5 min and centrifuged at 10,000 *g* for 10 min, and the supernatant was aspirated and discarded. The precipitated aluminum was washed twice with distilled water and then shaken vigorously with 2.5 ml of 0.4 N acetate. The mixture was centrifuged, and the clear supernatant was transferred to a small glass tube and lyophilised for 3 h, and the residue was dissolved in 0.5 ml of 0.2 N acetate. Then a 50 μl aliquot of this solution was injected into a liquid-chromatographic column (Hitachi 3011-C gel column, 2.6 × 250 mm, Tokyo, Japan). Materials were eluted with 0.1 M $KH_2PO_4$ containing 0.05% $H_3PO_4$ at a constant flow rate of 0.5 ml $min^{-1}$ at 45.0 ± 0.2°C. The effluent was mixed with the reagent for the trihydroxyindole reaction, consisting of 0.0075% potassium ferricyanide, 0.1% ascorbic acid, and 5 N sodium hydroxide. The resulting fluorescent products were examined with a highly sensitive spectro-fluorophotometer (Hitachi 650-10, Hitachi Ltd, Tokyo).

### *Statistical analysis*

Data were analysed by the Chi-square test or Fisher's exact probability test or by one-way analysis of variance with Dunn's multiple comparison (Miller, 1966; Siegel, 1956; Snedecor & Cochran, 1967). Data are given as means ± s.e. Results are reported as significant if the calculated *P* value is less than 0.05.

### Results

### *Incidence, number, histological type, and depth of involvement of gastric cancers*

Total amount of MNNG ingested for 25 weeks per rat was 170 ± 12 mg.

Five rats in each group were killed in week 30 for determination of the labelling index of the gastric mucosa. One rat in Group 2 and two rats in Group 1 were killed before week 50 because they became moribund. No tumours were found in any of these animals, which were excluded from the effective numbers.

The incidences, numbers, histological types and depths of involvement of gastric cancers are summarised in Table I. In Group 1 (olive oil only), gastric cancers were found in 6 (33%) of 18 rats examined, and the average number of

**Table I** Incidence, number, histological type, and depth of involvement of gastric cancers in MNNG-treated rats

| Group No. | Treatment[a] | Body Weight (g) Week 26 | Body Weight (g) Week 52 | Effective no. | No. of rats with gastric cancer (%) | No. of gastric cancers | No. of gastric cancers per rat | Histology (%) Very well-differentiated | Histology (%) Well-differentiated | Depth of involvement (%) Subnucosal layer | Depth of involvement (%) Muscle layer or deeper |
|---|---|---|---|---|---|---|---|---|---|---|---|
| 1 | Olive oil | 338 ± 6 | 396 ± 7 | 18 | 6 (33) | 7 | 0.4 ± 0.1 | 6 (86) | 1 (14) | 7 (100) | 0 (0) |
| 2 | Bromocriptine 1 mg $kg^{-1}$ | 334 ± 7 | 384 ± 11 | 19 | 15 (79)[c] | 21 | 1.1 ± 0.2[d] | 19 (90) | 2 (10) | 19 (90) | 2 (10) |
| 3 | Bromocriptine 2 mg $kg^{-1}$ | 324 ± 8 | 383 ± 5 | 20 | 15 (75)[b] | 19 | 1.0 ± 0.2[b] | 16 (84) | 3 (16) | 17 (89) | 2 (11) |

[a]Treatment regimens: Olive oil, 1 ml $kg^{-1}$ of the vehicle, olive oil, was given s.c. every other day after MNNG treatment for 25 weeks; Bromocriptine 1 or 2 mg $kg^{-1}$, 1 or 2 mg $kg^{-1}$ of bromocriptine in depot form was given s.c. every other day after MNNG treatment for 25 weeks. [b-d]Significantly different from the value for Group 1: [b]$P < 0.05$; [c]$P < 0.02$; [d]$P < 0.01$.

gastric cancers per rat was 0.4 ± 0.1. The incidence and the average number of gastric cancers per rat was 79% and 1.1 ± 0.2, respectively, in Group 2 (bromocriptine at 1 mg $kg^{-1}$), and 75% and 1.0 ± 0.2, respectively, in Group 3 (bromocriptine at 2 mg $kg^{-1}$): the differences were significantly significant from the values for Group 1.

As shown in Table I, all tumours induced in the glandular stomach were identified histologically as adenocarcinomas. The distributions of the different histological types of adenocarcinomas were not significantly different in the three groups. No poorly differentiated adenocarcinomas were found in this series. There was also no significant differences in the depths of involvement of gastric cancers in the three groups. All cancers were found in the antral mucosa, with no metastasis in any rat.

*Tissue norepinephrine, labelling index, antral pH, and serum gastrin*

Table II summarises data on the norepinephrine concentrations in the gastric wall, labelling indices of the gastric mucosa, antral pH's, and serum gastrin levels in the three groups in week 30 and/or week 52. The tissue norepinephrine concentrations in the fundic and antral portions of the stomach were slightly, but not significantly, higher in Groups 2 and 3 (bromocriptine at 1 and 2 mg $kg^{-1}$) than in Group 1 (olive oil). Epinephrine was not detected in any sample obtained from the gastric wall. In weeks 30 and 52, the labelling indices of the antral, but not the fundic mucosa in the bromocriptine-treated Groups 2 and 3 were significantly higher than that in Group 1 and the antral pH in Group 3 was significantly elevated. There were no significant differences in the serum gastrin levels in the three different groups.

The present study showed that the dopamine agonist bromocriptine promoted gastric carcinogenesis induced by MNNG in Wistar rats. Treatment of rats with bromocriptine in depot form after 25 weeks of oral treatment with MNNG resulted in a significant increase in the incidence and number of gastric cancers in week 52.

The mechanisms of the effect of bromocriptine are not fully understood, but several possible mechanisms may be considered. One possibility involves a pharmacological effect related to serotonin. Some data derived from animal studies suggest that the serotonin and dopamine system interact (Gershon & Baldessarini, 1980). Autoradiographic studies in rat brain have provided anatomical bases for possible interactions at axo-axonal synaptic connections between serotonin neurons and dopamine nigrostriated and mesolimbic circuits (Jenner *et al.*, 1983). Tutton (1974) found that small amounts of serotonin increased crypt cell renewal in the jejunum of the rat.

A second possibility is an effect of dopamine on the parasympathetic nervous system. Nishikawa *et al.* (1987) reported that dopamine inhibits vagally induced gastric acid secretion through an alpha-2 adrenoceptor-mediated mechanism. However, in studies on the involvement of dopamine receptors in cholinergic transmission in guinea pig stomach, Kusunoki *et al.* (1985) found that dopamine inhibited transmural stimulation-induced $^{3}H$-acetylcholine release and concluded that the release of acetylcholine from postganglionic cholinergic neurons is probably required through dopamine receptors that were antagonised by $D_2$ antagonist. Recently, we examined the role of the parasympathetic nervous system in the development of gastric cancers induced by MNNG, and found that prolonged injections of parasympatholytic atropine in depot form every other day resulted in a significant increase in the number of gastric cancers per rat (Tatsuta *et al.*, 1989). These findings indicate that the parasympathetic nervous system is closely involved in the development of gastric cancers.

A third possibility is an effect on the norepinephrine level in the gastric mucosa. Steardo *et al.* (1986) observed significant decrease of plasma norepinephrine in normal or hypotensive subjects after chronic administration of bromocriptine. Baksi *et al.* (1986) measured the adrenal catecholamine concentration in male rats after s.c. treatment with bromocriptine and haloperidol for 10 days. They found that bromocriptine-treatment resulted in significant increases in the dopamine, norepinephrine and epinephrine contents, but that haloperidol treatment had little or no influence on their contents. Hrbek *et al.* (1986) reported that application of bormocriptine for 10 days significantly decreased the dopamine content, but not the norepinephrine content of the brain of female rats. In the present work, we found that prolonged administration of bromocriptine slightly, but not significantly, elevated the norepinephrine concentration in gastric wall.

In the present study administration of the dopamine agonist bromocriptine every other day led to significant increase in the incidence and number of gastric cancers and in the labelling indices of the gastric antral mucosa. Although without work with dopaminergic antagonists it seems premature to ascribe these effects on tumour incidence entirely to its action on the dopaminergic system, these findings indicate that the development of gastric cancers is regulated by a dopaminergic mechanism, and that this mechanism may be closely related to an effect in increasing proliferation of antral epithelial cells.

### References

ANTON, A.H. & SAYRE, D.F. (1962). A study of the factors affecting the aluminum oxide-trihydroxyindole procedure for the analysis of catecholamines. *J. Pharmacol. Exp. Ther.*, **138**, 360.

BAKSI, S.N., HUGHES, M.J. & STRAHLENDORF, H.K. (1986). Adrenal catecholamine concentration after chronic treatment with bromocriptine and haloperidol. *J. Pharm. Pharmacol.*, **38**, 774.

BRODDE, O.-E. (1982). Vascular dopamine receptors: demonstration and characterization by *in vitro* studies. *Life Sci.*, **31**, 289.

BUENO, L., FARGEAS, M.J., FIORAMONTI, J. & HONDE, C. (1984) Effects of dopamine and bromocriptine on colonic motility in dog. *Br. J. Pharmac.*, **82**, 35.

EASTWOOD, G.L. & QUIMBY, C.F. (1983). Effect of chronic cimetidine ingestion on fundic and antral epithelial proliferation in the rat. *Dig. Dis. Sci.*, **28**, 61.

GERSHON, S.C. & BALDESSARINI, R.I. (1980). Motor effects of serotonin in the central nervous system. *Life Sci.*, **27**, 1435.

GRATZNER, H.G. (1982). Monoclonal antibody to 5-bromo- and 5-iododeoxyuridine: a new reagent for detection of DNA replication. *Science* 218, 474.

GULDVOG, I., LINNESTAD, P., SCHRUMPF, E. & BERSTAD, A. (1984). Dopaminergic and adrenergic influence on gastric acid and pepsin secretion stimulated by food. The role of vagal innervation. *Scand. J. Gastroenterol.*, **89**(Suppl.), 113.

HERNANDEZ, D.E., MASON, G.A., WALKER, C.H. & VALENZUELA, J.E. (1987). Dopamine receptors in human gastrointestinal mucosa. *Life Sci.*, **41**, 2717.

HRBEK, J., RYPKA, M. & HRBEK, J.Jr. (1986). Effect of bromocriptine on norepinephrine and dopamine levels in female rat brain. *Acta Univ. Palacki. Olomuc. Fac. Med.*, **115**, 99.

JENNER, P., SHEEHY, M. & MARSDEN, C.D. (1983). Noradrenaline and 5-hydroxytryptamine modulation of brain dopamine function: implications for the treatment of Parkinson's disease. *Br. J. Clin. Pharmacol.*, **5**(suppl.), 2779.

KUSUNOKI, M., TANIYAMA, K. & TANAKA, C. (1985). Dopamine regulation of [$^3$H]acetylcholine release from guinea-pig stomach. *J. Pharmacol. Exp. Ther.*, **234**, 713.

MILLER, R.G. Jr. (1966). *Simultaneous Statistical Inference*. McGraw-Hill: New York.

MORSTYN, G., HSU, S.M., KINSELLA, T., GRATZNER, H., RUSSO, A. & MITCHELL, J.B. (1983). Bromodeoxyuridine in tumors and chromosome detected with a monoclonal antibody. *J. Clin. Invest.*, **72**, 1844.

NISHIKAWA, H., YOKOTANI, K. & FUJIWARA, M. (1987). Catecholamine receptors involved in the inhibitory effects of dopamine on vagally stimulated gastric acid secretion and mucosal blood flow in rats. *J. Pharmacol. Exp. Ther.*, **240**, 966.

ODAIBO, S.K., CHEY, W.Y. & LEE, K.Y. (1983). Presence and role of local dopamine receptors on antral motility in the isolated perfused rat stomach. *Gastroenterology (abstract)*, **84**, 1262.

RATTAN, S. & GOYAL, R.K. (1976). Effect of dopamine on the esophageal smooth muscle in vivo. *Gastroenterology*, **70**, 377.

SCEMAMA, J.L., RUELLAN, C., CLERC, P., CLEMENTE, F. & RIBET, A. (1984). Dopamine receptors in a human colonic cancer cell line (HT29). Some receptor-related biological effects of dopamine. *Int. J. Cancer*, **34**, 675.

SIEGEL, S. (1956). *Nonparametric Statistics for the Behavioral Sciences*. McGraw-Hill: New York.

SNEDECOR, G.W. & COCHRAN, W.G. (1967). *Statistical Methods*. The Iowa State University Press: Ames, IA.

STEARDO, L., DI STASIO, E., BONUSO, S. & MAJ, M. (1986). The effect of bromocriptine on plasma catecholamine concentrations in normal volunteers. *Eur. J. Clin. Pharmacol.*, **29**, 713.

STEFANINI, M., DE MARTINO, C. & ZAMBONI, L. (1967). Fixation of ejaculated spermatozoa for electron microscopy. *Nature*, **216**, 173.

TADA, T., KODAMA, T., WATANABE, S., SATO, Y. & SHIMOSATO, Y. (1985). Cell kinetic studies by the use of anti-bromodeoxyuridine monoclonal antibody and their clinical application. *Igaku-no-ayumi*, **135**, 510.

TATSUTA, M., ITOH, T., OKUDA, S., TAMURA, H. & YAMAMURA, H. (1977). Effect of fundusectomy on serum and antral gastrin level in rats. *Gastroenterology*, **72**, 78.

TATSUTA, M., BABA, M. & ITOH, T. (1983). Increased gastrin secretion in patients with pheochromocytoma. *Gastroenterology*, **84**, 920.

TATSUTA, M., IISHI, H., YAMAMURA, H., BABA, M., YAMAMOTO, R. & TANIGUCHI, H. (1988). Effect of cimetidine on inhibition by tetragastrin of carcinogenesis induced by N-methyl-N'-nitro-N-nitrosoguanidine in Wistar rats. *Cancer Res.*, **48**, 1591.

TATSUTA, M., IISHI, H. & BABA, M. (1989). Inhibition by neostigmine and isoproterenol and promotion by atropine of experimental carcinogenesis in rat stomach by N-methyl-N'-nitro-N-nitrosoguanidine. *Int. J. Cancer*, **44**, 188.

TUTTON, P.J.M. (1974). The influence of serotonin on crypt cell proliferation in the jejunum of the rat. *J. Anat.*, **118**, 389.

VALENZUELA, J.E. (1976). Dopamine as a possible neurotransmitter in gastric relaxation. *Gastroenterology*, **71**, 1019.

VAN NEUTEN, J.M. (1980). Is dopamine an inhibitory modulator of gastric motility? *Trends Pharmacol. Sci.*, **9**, 233.